# Enrichment of ER tubule-derived microsomes by differential centrifugation and immunoprecipitation

**DOI:** 10.52601/bpr.2023.230031

**Published:** 2024-04-30

**Authors:** Yiduo Liu, Junjie Hu, Bing Yan

**Affiliations:** 1 National Laboratory of Biomacromolecules, CAS Center for Excellence in Biomacromolecules, Institute of Biophysics, Chinese Academy of Sciences, Beijing 100101, China; 2 University of Chinese Academy of Sciences, Beijing 101408, China

**Keywords:** Endoplasmic reticulum, Microsome, Differential centrifugation, Immunoprecipitation

## Abstract

The endoplasmic reticulum (ER) is an essential component of the endomembrane system in eukaryotes and plays a crucial role in protein and lipid synthesis, as well as the maintenance of calcium homeostasis. Morphologically, the ER is composed primarily of sheets and tubules. The tubular ER is composed of a network of tubular membrane structures, each with diameters ranging from 30 to 50 nanometers. In recent years, there has been in-depth research on the molecular mechanisms of membrane shaping and membrane fusion of the tubular ER. However, there is still limited understanding of the specific physiological functions of the tubular ER. Here, we report a protocol that combines differential centrifugation and immunoprecipitation to specifically enrich microsomes originating from the tubular ER in yeast. The ER tubule-derived microsomes can be further used for proteomic and lipidomic studies or other biochemical analyses.

## INTRODUCTION

The endoplasmic reticulum (ER) is a complex organelle in eukaryotic cells and is involved in many cellular physiological processes. It is a continuous membrane system consisting of networks of tubules and sheets. ER tubules have a diameter of approximately 30 nm in yeast and 50 nm in mammalian cells (Hu *et al.* 2011). These tubular structures are highly dynamic, constantly elongating, retracting, and fusing into networks (Griffing 2010). Previous studies have demonstrated a class of reticulon homology domain (RHD) containing integral membrane proteins, such as RTNs and DP1/Yop1p, which are responsible for generating and maintaining high membrane curvature (Hu *et al.* 2008; Shibata *et al.* 2008; Voeltz *et al.* 2006; Xiang *et al.* 2023). Membrane fusion between ER tubules is mediated by a class of dynamin-like GTPases, including atlastin (ATL) in mammalian cells and sey1p in yeast (Anwar *et al.* 2012; Bian *et al.* 2011; Hu *et al.* 2009; Yan *et al.* 2015). ER tubules play central roles in forming membrane contact sites (MCSs) with other organelles (Guillen-Samander and De Camilli 2023; Phillips and Voeltz 2016). In the MCSs, the ER tubules facilitate the transfer of proteins, lipids, and other signal molecules with the tethered organelles. Aberrant expression of ER tubule-forming proteins results in abnormal ER morphology, affecting the physiological functions of the cell. Mutations of ATL1 in humans result in defective neurite outgrowth and cause hereditary spastic paraplegia (HSP) (Li *et al.* 2017; Salinas *et al.* 2008). RHD3, the homolog of ATL in *A. thaliana*, is crucial for root hair development (Zhang *et al.* 2013).

**Figure 1:**
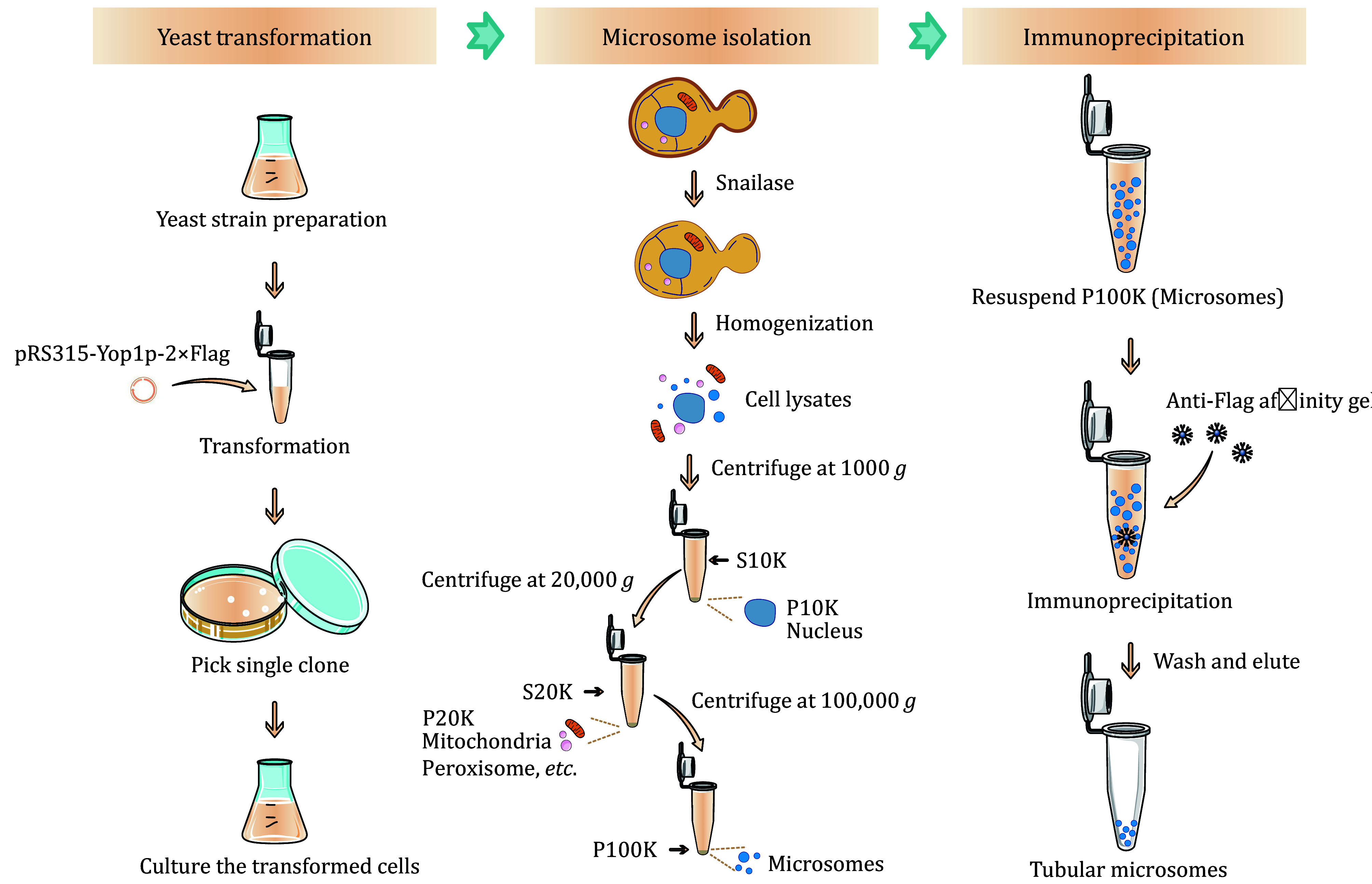
The workflow of the enrichment of ER tubule-derived microsomes

ER fractions in the form of microsomes are isolated from cell homogenates by differential centrifugation and are widely used for *in vitro* studies of the biological functions of the ER (Kriechbaumer 2018). We previously isolated ER tubule-derived microsomes and identified 79 tubule-enriched proteins through quantitative proteomics. These proteomic profiles suggest that the tubular ER network may be involved in lipid metabolism, membrane contacts, and stress sensing (Wang *et al.* 2017). Here, we provide a detailed description of the protocol for isolating the ER tubule-derived microsomes via immunoprecipitation against a tubule-specific marker from yeast cell microsomes (Fig. 1). The purified ER tubule-derived microsomes can be further utilized for proteomics or lipidomics analyses to identify the ER tubule-enriched proteins or lipids and may be helpful for understanding the biological functions of tubular ER. This method is also adaptable for the isolation and purification of other organelles or subcellular structures.

## STEP-BY-STEP PROCEDURE

### Step 1: Transformation and protein expression validation in yeast [TIMING 7–10 d]

#### Step 1.1: Preparation

Step 1.1.1: Inoculate BY4741 wild-type strain into 5 mL of fresh YPD medium and incubate at 30 °C overnight with shaking at 220 r/min.

Step 1.1.2: Dilute the cells from the above culture into fresh YPD medium until OD_600_ = 0.1 and incubate at 30 °C to reach an OD_600_ of 0.8 (about 7–8 h) with shaking at 220 r/min.

#### Step 1.2: Transform yeast strain preparation

Step 1.2.1: Pellet the cells gently from a 1.5 mL culture of wild-type strain BY4741 in YPD medium by centrifugation at 5000 *g* for 3 min at room temperature.

Step 1.2.2: Discard the supernatant and resuspend the cell pellet in 1 mL 0.1 mol/L LiAc solution.

Step 1.2.3: Centrifuge cells at 5000 *g* for 3 min at room temperature.

Step 1.2.4: Discard supernatant and resuspend the cell pellet in 100 μL 0.1 mol/L LiAc solution.

#### Step 1.3: Introduce target plasmid

Step 1.3.1: Add 5 μL salmon essence DNA (heat at 95 °C for 10 min, make it completely denaturated, quickly place it on ice for 2 min), vortex, and mix.

Step 1.3.2: Add 0.1 µg of plasmid DNA (pRS315-Yop1p-2×Flag) and mix. Add 700 μL PEG4000, vortex, and mix.

Step 1.3.3: Incubate at 30 °C for 30 min with shaking at 200 r/min.

Step 1.3.5: Heat shock for 25 min in a 42 °C water bath and add 700 µL of YPD medium.

Step 1.3.6: Incubate at 30 °C for 60 min with shaking at 200 r/min.

Step 1.3.7: Centrifuge cells for 3 min at 5000 *g* at room temperature and remove the supernatant.

Step 1.3.8: Resuspend cells in 150 μL of sterile deionized water and plated on a selective medium that will select for the desired transformants. Incubate plates upside-down at 30 °C until colonies appear (generally 2–5 days).

#### Step 1.4: Validation

Step 1.4.1: After incubating the transformed yeast strain in a 30 °C incubator for 2–5 days, use a sterile tip to pick up four monoclonal strains (2–3 mm diameter) stretched out on the corresponding nutrient-deficient selective medium.

**[TIP]** Vigorously vortex the medium for ~1 min to thoroughly disperse the cells.

Step 1.4.2: Incubate at 30 °C for three days with shaking at 220 r/min to achieve OD_600_ = 1.

Step 1.4.3: Collect 2 mL of culture and centrifuge at 5000 *g* for 3 min at room temperature.

Step 1.4.4: Discard the supernatant and thoroughly resuspend the cell pellets in 1 mL sterile 0.5 mol/L NaOH solution for 10 min at room temperature.

Step 1.4.5: Centrifuge cells for 3 min at 5000 *g* at room temperature and remove the supernatant.

Step 1.4.6: Add 30 µL 2× SDS loading buffer and boil at 65 °C for 10 min.

Step 1.4.7: Centrifuge at 20,000 r/min and collect the supernatant for Western blot.

### Step 2: Yeast microsome preparation [TIMING 3 h]

#### Step 2.1: Preparation of spheroplasts

**[TIP]** The sample should not be frozen during the whole process, otherwise the microsomal vesicles will burst.

Step 2.1.1: Culture the transformed yeast at 30 °C to an OD_600_ of 1.

Step 2.1.2: Collect 10 mL of culture and centrifuge at 5000 *g* for 3 min at 4 °C.

Step 2.1.3: Discard the supernatant and wash the cell pellets twice with 1.5 mL 0.05 mol/L EDTA (pH 8.0). Centrifuge at 5000 *g* for 3 min at 4 °C and collect the cell pellet.

Step 2.1.4: Resuspend the cell pellet in 1 mL ETB buffer.

Step 2.1.5: Incubate at 30 °C for 30 min with shaking at 200 r/min.

Step 2.1.6: Centrifuge cells for 3 min at 5000 *g* at 4 °C and remove the supernatant.

Step 2.1.7: Resuspend the cell pellet in 1.5 mL 2% Snailase buffer at 30 °C for 1 h.

Step 2.1.8: Centrifuge cells for 3 min at 5000 *g* at 4 °C, remove the supernatant, and collect the cell pellet.

**[TIP]** It is recommended to use a 5 mL centrifuge tube for treatment and seal the centrifuge tube with sealing film. At this point, the cell wall of the yeast cell has been digested by the snail enzyme and the yeast has become spheroplasts.

Step 2.1.9: Wash the cell pellet with 1.5 mL Sorbitol buffer twice and centrifuge cells for 3 min at 5000 *g* at 4 °C to collect the spheroplasts.

**[TIP]** Gently resuspend the yeast after digesting the cell wall.

#### Step 2.2: Microsome preparation

Step 2.2.1: Resuspend and swell the spheroplasts in 1 mL lysis buffer 1 for 15 min on ice.

**[TIP]** Pre-cool the homogenizer before starting the procedure. Homogenization and the following steps must be carried out at 4 °C (*i*.*e*., in a cold room) to minimize the activation of proteases and phospholipases.

Step 2.2.2: Homogenize the cell suspension with 35 strokes in a tight-fitted Dounce homogenizer.

**[TIP]** Carefully observe each homogenization step, though it is difficult to determine the extent of cell disruption by eye. Examine the scraped cell suspension under the light microscope and then reexamine the homogenate through the light microscope. When the cells are well homogenized, there will be an apparent difference: The cell suspension will appear granular, whereas the homogenate will appear milky and opalescent. In addition, when the cells are disrupted, the homogenate begins to foam during homogenization. This change may be used as an indication of cell disruption. Naturally, the extent of cell disruption may also be examined by a phase contrast microscope.

**[CRITICAL STEP]** Hold the tube of the Dounce homogenizer on the table at a slight angle. Insert the glass pestle into the tube slowly until the tip reaches the surface of the cell suspension, and then push the pestle down and pull it up, applying force with the pestle on one side of the tube. Once up and down is considered one stroke.

**[?TROUBLESHOOTING]**

Step 2.2.3: Centrifuge the homogenate in a refrigerated centrifuge at 4 °C at 1000 *g* for 5 min and collect the cell suspension. Resuspend the pellets in 500 μL of lysis buffer 1 and homogenize the suspension with 35 strokes. Mix the first and second homogenate.

Step 2.2.4: Centrifuge the homogenate in a refrigerated centrifuge at 4 °C at 1000 *g* for 5 min, collect the cell suspension, and discard the pellet (containing unbroken cells and nuclei).

Step 2.2.5: Centrifuge the suspension at 20,000 *g* (rotor FA-45-24-11, Eppendorf) at 4 °C for 30 min, collect the cell suspension, and discard the pellet (containing lysosomes and mitochondria).

Step 2.2.6: Further centrifuge the obtained supernatant at 100,000 *g* for 40 min at 4 °C (rotor TLA 100.3, Beckman) to isolate the microsome fraction (P100K pellet) and cytosolic fraction (supernatant).

**[TIP]** Store a small amount of fraction from each step for further investigations (*e*.*g*., Western blot) in a 1.5-mL tube. Freeze at −80 °C if not used immediately.

### Step 3: Immunoprecipitation of tubular ER [TIMING 3h]

#### Step 3.1: Prepare beads for immunoprecipitation

Step 3.1.1: Add 40 μL of 1:1 suspension of the Anti-Flag affinity gel.

**[TIP]** It is recommended to transfer the gel using a 200-μL “automatic” pipette with the cut-out end of the tip.

Step 3.1.2: Pellet the resin by a short spin (12,000 *g*, 30 s) and remove the supernatant.

Step 3.1.3: Wash the resin twice with 1 mL PBS, and then wash the resin with 1 mL lysis buffer.

#### Step 3.2: Immunoprecipitation

Step 3.2.1: To isolate Yop1p-containing microsomes, resuspend P100k in 700 μL of lysis buffer 2 and place on ice for 15 min.

Step 3.2.2: After incubation, add supernatant to the settled resin.

Step 3.2.3: Incubate for 2 h on an orbital shaker at 4 °C. Shaking must be vigorous enough to suspend the resin.

Step 3.2.4: The affinity gel with bond vesicles was pelleted at 800 *g* (rotor FA-45-24-11, Eppendorf) at 4 °C for 2 min.

Step 3.2.5: Wash the resin twice with 1 mL lysis buffer 2. After the final wash, aspirate the supernatant and leave ~10 μL above the beads.

**NOTE:** For Western blot analysis, boil the pellet directly in 2× SDS-PAGE sample loading buffer. For mass spectrometric analysis, solubilize the pellet in 0.1 mol/L ammonium bicarbonate buffer containing 0.1% RapiGest SF and boil for 20 min. The supernatant is collected for mass spectrometry. For the Percoll gradient assay, resuspend the pellet in lysis buffer 1 containing Flag peptides (1 μg/mL) to elute the bonding vesicles.

### Step 4: Verification of the isolated microsome

#### Step 4.1: Percoll gradient assay [TIMING 2 h]

Step 4.1.1: Prepare 30% Percoll from a 100% Percoll solution by mixing 100% Percoll with 1× PBS at a ratio of 3:7 (volume).

Step 4.1.2: Pipette 2 mL of the 30% Percoll solution into a centrifugation tube.

(1) Carefully layer total microsomes (P100k) resuspended in PBS or samples eluted from the immunoprecipitated beads (200 μL) on top of the Percoll solution.

(2) Centrifuge at 95,000 *g* (rotor TLS55, Beckman) at 4 °C for 40 min.

Step 4.1.3: After centrifugation, fractionate the gradient into 21 tubes, 100 μL per fraction.

Step 4.1.4: Analyze the samples by Western blotting and quantify them with ImageJ.

#### Step 4.2: Electron microscopy [TIMING 5 h]

Step 4.2.1: Perform negative-stain EM with 2% uranyl acetate solubilized in deionized water.

Step 4.2.2: Elute immunoprecipitated microsomes (see Step 3.2.5) using 1 μg/mL Flag peptide.

Step 4.2.3: Concentrate the eluate ~20-fold by centrifugal filters (Merck Millipore).

Step 4.2.4: Place 5 μL of sample solution onto a glow-discharged carbon-coated copper grid for 1 min.

Step 4.2.5: Remove excess sample using filter paper and wash the grids with one drop of deionized water. Stain with one drop of fresh 2% uranyl acetate for 40 s.

Step 4.2.6: Collect images at room temperature using a HITACHI HT7700 transmission electron microscope.

**[?TROUBLESHOOTING]**

Troubleshooting advices are listed in Table 1.

**Table 1:** Troubleshooting table

Step	Problem observed	Possible reason	Solutions
2.2.2	Low amount of isolated microsome pellet.	(1) Not enough material was used for isolation. (2) Homogenization was insufficient.	Use more cells for isolation. Homogenize longer. Check cell disruption under microscope.

## MATERIALS AND EQUIPMENT

### Reagents

• PBS (Vivacell, Cat. No. C3580-0500)

• Tris Base (Sigma, Cat. No. V900483)

• Percoll (GE, Cat. No. V900483)

• Protease inhibitor cocktail (Biomake, Cat. No. B14012)

• Tunicamycin (Abcam, Cat. No. ab120296)

• Agarose (LabLead, Cat. No. AG0100)

• Snailase (Sangon, Cat. No. A600870-0005)

• Anti-Flag affinity gel (Sigma, Cat. No. F2426)

• 0.1% RapiGest SF (Waters, Cat. No. 186001861)

• YPD broth (Sangon, Cat. No. A507022-0250)

• Salmon Sperm DNA Solution (ThermoFisher, Cat. No. 15632011)

### Equipment

• Dounce tissue homogenizer (Kimble, Cat. No. K885300-0002)

• 1.5-mL polypropylene tube (Beckman, Cat. No. 357448)

• Rotor TLA 100.3 (Beckman, Cat. No. 349481)

• Optima MAX-XP tabletop ultracentrifuge (Eppendorf, Cat. No. 5405000697)

• Centrifuge 5425 (Beckman, Cat. No. 393315)

### Reagent setup

• 5 mol/L NaCl stock

• 1 mol/L Tris-Cl (pH 8.0) stock

• 0.5 mol/L EDTA (pH 8.0)stock

• 0.2 mol/L Na_2_HPO_4_ stock

• 2 mol/L sorbitol stock

• 0.2 mol/L sodium citrate stock

• 0.1 mol/L triethanolamine stock

• 0.1 mol/L ammonium bicarbonate buffer

• LiAc solution: 10 mmol/L Tris-Cl, 1 mmol/L EDTA, 100 mmol/L lithium acetate

• TE buffer (10 mmol/L Tris, 1 mmol/L EDTA, pH 8.0). To prepare 50 mL of TE buffer, mix 500 μL of 1 mol/L Tris (pH 9.0) and 100 μL of 0.5 mol/L EDTA, adjust the pH to 8.0 using HCl, bring the solution to a final volume of 50 mL with ddH_2_O, and store at 4 °C.

• ETB buffer (0.05 mol/L EDTA, 0.1 mol/L Tris-Cl, 2.5% BME, pH 7.4). To prepare 50 mL of ETB buffer, mix 5 mL of 500 mmol/L EDTA, 5 mL of 1 mol/L Tris, and 1.25 μL of 100% BME and bring the solution to a final volume of 50 mL with bi-distilled water and store at 4 °C. This buffer needs to be prepared fresh the day before or on the day of the experiment.

• Sorbitol buffer (1 mol/L sorbitol, 0.02 mol/L sodium citrate, 0.1 mol/L EDTA, 0.02 mol/L Na_2_HPO_4_, pH 5.8). To prepare 50 mL of sorbitol buffer, mix 25 mL 2 mol/L sorbitol, 5 mL 200 mmol/L sodium citrate, 10 mL 0.5 mol/L EDTA, and 10 mL 0.2 mol/L Na_2_HPO_4_ and store at 4 °C.

• Snailase buffer: To prepare 100 mL of snailase buffer, dissolve 0.2 g of snailase in 100 mL sorbitol buffer and store at 4°C.

• Lysis buffer 1 (800 mmol/L sorbitol, 10 mmol/L triethanolamine, 1 mmol/L EDTA pH 8.0, and protease inhibitor cocktail). To prepare 50 mL of lysis buffer 1, mix 20 mL 2 mol/L sorbitol, 500 μL 1 mol/L triethanolamine, and 100 μL 0.5 mol/L EDTA, add the appropriate protease inhibitor cocktail according to the manual, bring the solution to 50 mL with bi-distilled water, and store at 4 °C. This buffer needs to be prepared fresh the day before or on the day of the experiment.

• Lysis buffer 2 (800 mmol/L sorbitol, 150 mmol/L NaCl, 10 mmol/L triethanolamine, 1 mmol/L EDTA pH 8.0, and protease inhibitor cocktail). To prepare 50 mL of lysis buffer 1, mix 20 mL 2 mol/L sorbitol, 1.5 mL 5 mol/L NaCl, 500 μL 1 mol/L triethanolamine, and 100 μL 0.5 mol/L EDTA, add the appropriate protease inhibitor cocktail according to the manual, bring the solution to 50 mL with bi-distilled water, and store at 4 °C. This buffer needs to be prepared fresh the day before or on the day of the experiment.

## Conflict of interest

Yiduo Liu, Junjie Hu and Bing Yan declare that they have no conflict of interest.
